# Impacts of breed type and vaccination on *Teladorsagia circumcincta* infection in native sheep in Gran Canaria

**DOI:** 10.1186/s13567-019-0646-y

**Published:** 2019-04-27

**Authors:** Jorge F. González, Julia N. Hernández, Cynthia Machín, Tara Pérez-Hernández, Harry W. Wright, Yolanda Corripio-Miyar, Daniel R. G. Price, Jacqueline B. Matthews, Tom N. McNeilly, Alasdair J. Nisbet

**Affiliations:** 1 Instituto Universitario Sanidad Animal y Seguridad Alimentaria, Facultad de Veterinaria, Universidad de Las Palmas de Gran Canaria, Arucas, Spain; 2 Moredun Research Institute, Pentlands Science Park, Edinburgh, EH26 0PZ UK

## Abstract

Vaccines and genetic resistance offer potential future alternatives to the exclusive use of anthelmintics to control gastrointestinal nematodes (GIN). Here, a *Teladorsagia circumcincta* prototype vaccine was administered to two sheep breeds which differ in their relative levels of resistance to infection with GIN. Vaccination of the more susceptible Canaria Sheep (CS) breed induced significant reductions in worm length and numbers of worm eggs in utero (EIU) when compared to control CS sheep. In the more resistant Canaria Hair Breed (CHB), although vaccination induced a reduction in all parasitological parameters analysed, differences between vaccinated and control sheep were not statistically significant. Such interactions between sheep breed and vaccination may allow better integrated control of GIN in future.

## Introduction, methods and results

One of the main limiting factors in sheep production worldwide is infection with gastrointestinal nematodes (GIN). In temperate regions, *Teladorsagia circumcincta* is amongst the most important of these parasites, both in terms of impact on animal health and welfare and in losses in productivity [1, 2]. Traditionally, these parasites have been controlled by regular administration of anthelmintics; however, the increasing prevalence of nematode resistance to these drugs requires alternative or complementary control methods [1, 3]. Sheep have been shown to develop protective immunity against a range of GIN following repeated exposure to the parasites [4, 5] and, amongst alternative control strategies being considered, those that exploit this phenomenon through selection of more genetically resistant animals [1] or by implementing effective vaccines [6] are attractive. Both strategies, vaccination and genetic resistance, are considered here.

Vaccines are considered an appealing alternative control measure for nematodes because they less likely to be subject to the development of parasite resistance and are environmentally friendly [7]. Although vaccination with parasite extracts has generated protection against GIN challenge in a number of trials, most recombinant versions of proteins identified in these fractions have failed to confer similar protection; this is a serious limitation for large-scale commercial vaccine production [5]. Recently, a vaccine based on eight recombinant antigens identified in *T. circumcincta* was shown to stimulate significant levels of protection in Texel-cross lambs [6] and also in ewes during the periparturient period [8] compared to matched challenged sheep. In both types of stock (lambs and ewes), significant reductions in faecal worm egg excretion were observed in vaccinates.

Several sheep breeds have been shown to be more resistant to GIN than other breeds [3]. The use of such resistant breeds offer a potential route to mitigate the effects of helminths in specific production systems. In the Canary Islands, for example, two local breeds of sheep are commonly farmed: the Canaria Hair Breed (CHB) and the Canaria Sheep (CS) breed. The CHB sheep have been shown to be more resistant than CS sheep when administered a single experimental infection of *Haemonchus contortus* [9]. Moreover, the former breed has been shown to be more resistant to a natural challenge infection comprising a mix of GIN [10]. Although both strategies are promising in terms of developing sustainable control methods for GIN with less reliance on the use of anthelmintics, neither is likely to entirely replace the use of anti-parasiticides [11]. Combining different alternative methods for worm control could be more effective than using either alone [12], and it would be of interest to explore, in reported resistant breeds, the additive, synergistic or antagonistic effect of vaccination to validate the combination of these control methods. This study tested this hypothesis by undertaking a comparative *T. circumcincta* vaccination and challenge study in the Canarian sheep breeds previously shown to be of different susceptibility to GIN.

Twenty-four CHB and CS lambs (4–5 months-old) were purchased, and, although no strongyle eggs were detected at purchase, they were dewormed with a subcutaneous application of ivermectin (Vectimax^®^, 0.2 mg/kg) and maintained in conditions designed to avoid helminth infection at the facilities of *Granja Experimental del Cabildo Insular de Gran Canaria* (Veterinary Faculty, Spain) until they were 6–7 months-old. Freedom from helminth infection was confirmed by further coprological testing just before the start of the trial. Animals were fed with a commercial pelleted sheep ration, with forage and water ad libitum throughout the experimental period. Animals were distributed randomly within breed in each experimental group (CS-vaccine; CS-control; CHB-vaccine; CHB-control). One lamb in the CHB-vaccine group died a few days after the start of the procedure from a post-traumatic renal haemorrhage.

**Figure 1:**
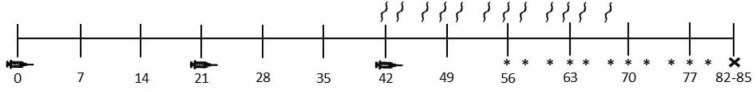
**Experimental protocol scheme.** The timeline represents days from the start of the experiment (first immunisation). The syringe icon represents each vaccine administration and the picture of larvae, the challenge inoculations. The “*” represents the collection of faeces sampled for faecal egg count analysis, and “x” denotes the time-point of euthanasia.

The recombinant vaccine was produced exactly as described previously [6]. Sheep in the two vaccinated groups were each injected subcutaneously with 400 µg of vaccine antigens incorporating 50 µg of each protein: cathepsin F-1 (Tci-CF-1), astacin-like metalloproteinase-1 (Tci-MEP-1), a 20 kDa protein of unknown function (Tci-ES20), activation-associated secretory protein-1 (Tci-ASP-1), a homologue of a protective antigen from *Ancylostoma caninum* (Tci-SAA-1), macrophage migration inhibitory factor-1 (Tci-MIF-1), calcium-dependent apyrase-1 (Tci-APY-1) and a TGF homologue (Tci-TGH-2). These were administered in 10 mg of the adjuvant, Quil A (Vax Saponin, Guinness Chemical Products Ltd). Seven of the proteins were phosphate buffered saline (PBS)-soluble and administered in a single injection with 5 mg Quil A in PBS. Tci-MEP-1 is insoluble in PBS and was formulated with 2 M urea in PBS with 5 mg Quil A. The preparations were injected separately at two sites behind the shoulder of each sheep. Three immunizations were administered intervals of 3 weeks. Sheep in each control group received three immunizations with the same concentrations and volumes of urea/PBS/Quil A at the same time as the vaccinates. On the day of the final immunization, an oral trickle third stage larval (L3) challenge was initiated; each sheep was given 2000 *T. circumcincta* L3, three times per week for 4 weeks as described previously [6] (Figure 1). For these infections, a UK-derived *T. circumcincta* strain (MTci2, Weybridge, UK) was used, from which all vaccine antigens were originally derived [6].

Faecal egg counts (FEC) were performed three times per week from 12 days after the start of larval challenge until the end of the experiment 4 weeks later. Cumulative FEC values were estimated for each group using the trapezoidal method for calculation of area under the curve (AUC, [13]). FEC data patterns were analysed by fitting generalised additive mixed models (GAMM) as described previously [6]. Differences in cumulative FEC and total worm burden were analysed using negative binomial models accounting for data over-dispersion.

**Figure 2:**
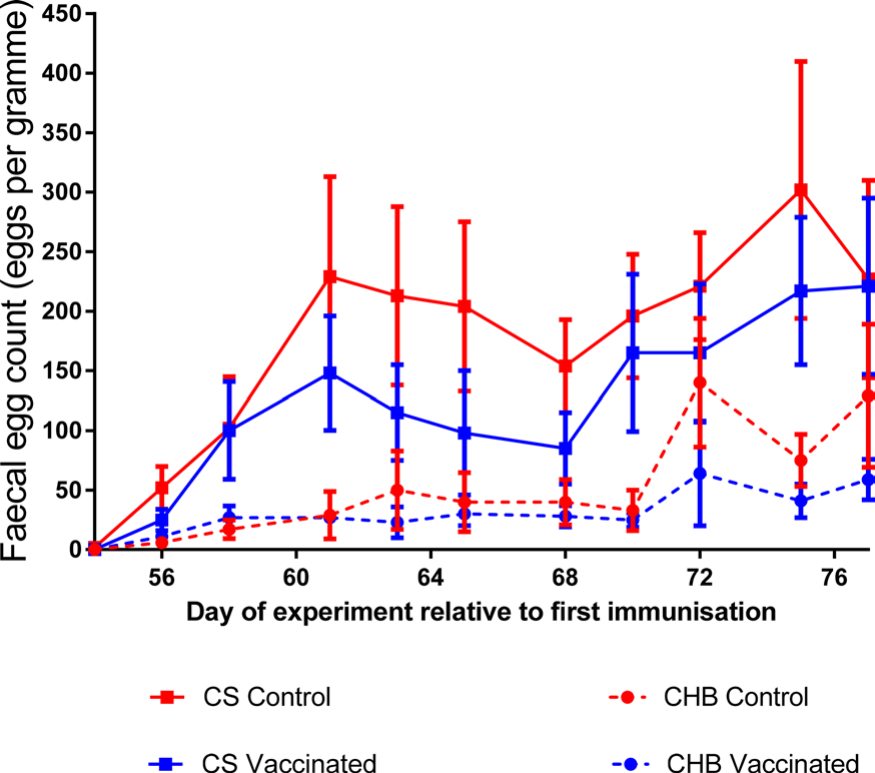
**Faecal egg counts measured after challenge infection in two sheep breeds vaccinated against** ***T. circumcincta.*** FEC are shown of sheep (CS, solid lines; CHB, dashed lines) challenged with 2000 *T. circumcincta* L3 three times per week for 4 weeks following immunization with an eight-protein cocktail in the context of Quil A (blue lines) or with Quil A only (red lines). Each data point represents the arithmetic mean FEC ± SEM (*n* = 12 for all groups except CHB vaccinated where *n* = 11).

**Figure 3:**
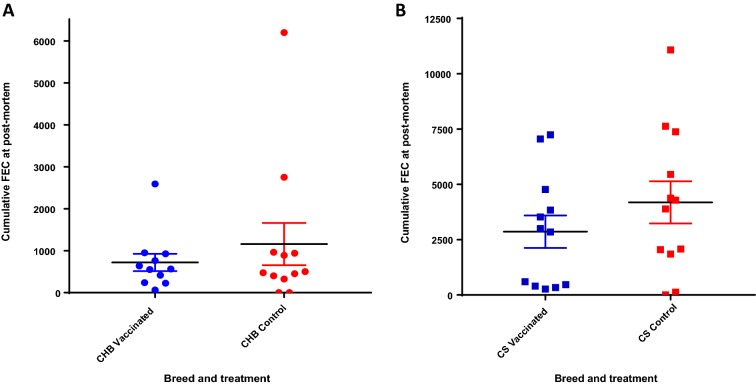
**Cumulative faecal egg counts after challenge infection in two sheep breeds vaccinated against** ***T. circumcincta.*** Cumulative FEC are shown of CHB sheep (“CHB” **A**) or CS (“CS” **B**) challenged with 2000 *T. circumcincta* L3 three times per week for 4 weeks following immunization with an eight-protein cocktail in the context of Quil A (CS or CHB vaccinated) or with Quil A only (CS or CHB Control). The mean of cumulative FEC is shown ± SEM (*n* = 12 for all groups except CHB vaccinated where *n* = 11).

Vaccinated and control sheep of both breeds began to excrete *T. circumcincta* eggs 14–16 days after the start of challenge (Figure 2). GAMM analysis identified a statistically significant effect of sheep breed on mean FEC over the time-course of the experiment, with significantly higher FEC in non-vaccinated CS than observed in non-vaccinated CHB (*p* = 0.005). In CS, FEC levels increased over time until 21 days after the start of challenge and, from 16 days post-challenge, vaccinated CS excreted substantially fewer eggs than CS control sheep at each time-point (Figure 2). GAMM analysis did not reveal a significant difference in mean FEC between vaccinated and unvaccinated CS (*p* = 0.118) or unvaccinated CHB sheep (*p* = 0.478) across the time-course. Mean cumulative FEC levels for CHB sheep for the duration of the challenge period were 1157 (± 504) eggs per gram (EPG) in controls and 720 (± 197) EPG in vaccinates, representing, overall, 38% lower cumulative FEC in the CHB vaccinates (*p* = 0.385; Figure 3A). Mean cumulative FEC for CS for the duration of the challenge period were 4181 (± 953) EPG in control sheep and 2860 (± 738) EPG in vaccinates, representing, overall, 32% lower mean cumulative FEC in CS vaccinates compared to the CS control lambs (*p* = 0.427; Figure 3B). Comparing the average cumulative FEC between control sheep of the two breeds, CS had, on average, 72% higher cumulative FEC levels than the CHB controls (*p* = 0.038).

**Figure 4:**
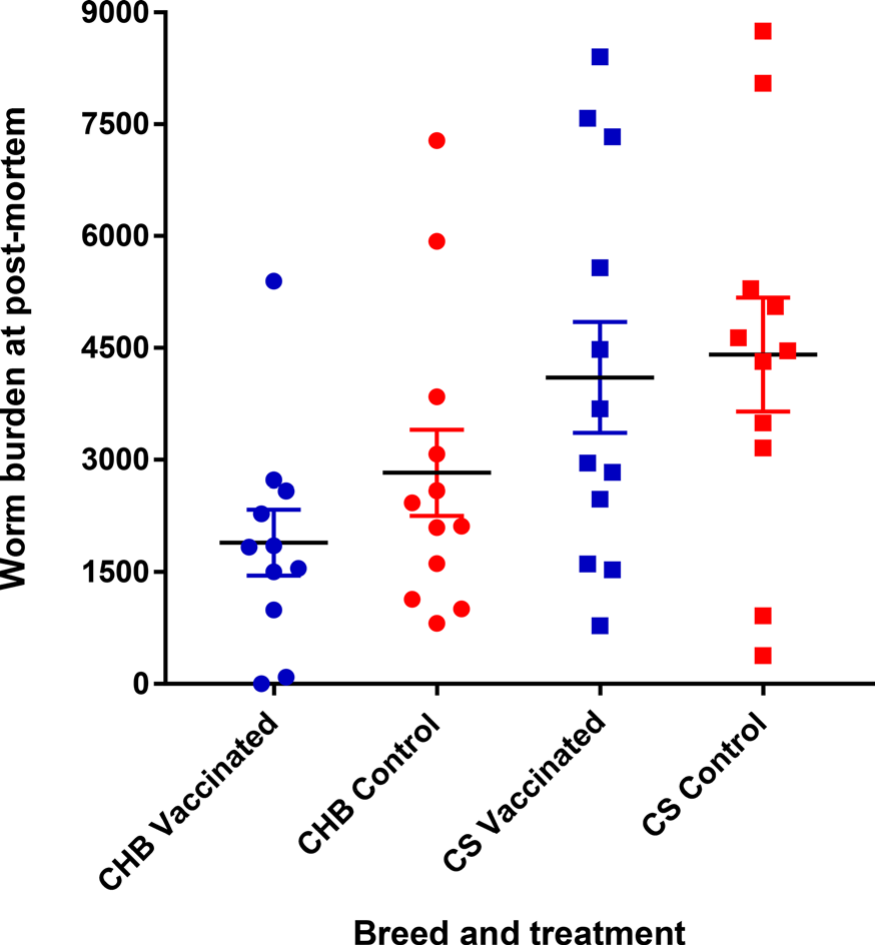
**Total worm burdens after challenge infection in two sheep breeds vaccinated against** ***T. circumcincta.*** Worm burdens are shown of sheep (CHB, circles; CS, squares) challenged with 2000 *T. circumcincta* L3 three times per week for 4 weeks following immunization with an eight-protein cocktail in the context of Quil A (blue symbols) or with Quil A only (red symbols). The mean worm burden is shown ± SEM (*n* = 12 for CS vaccinated and CHB control and in CS control and CHB vaccinated where *n* = 11).

**Figure 5:**
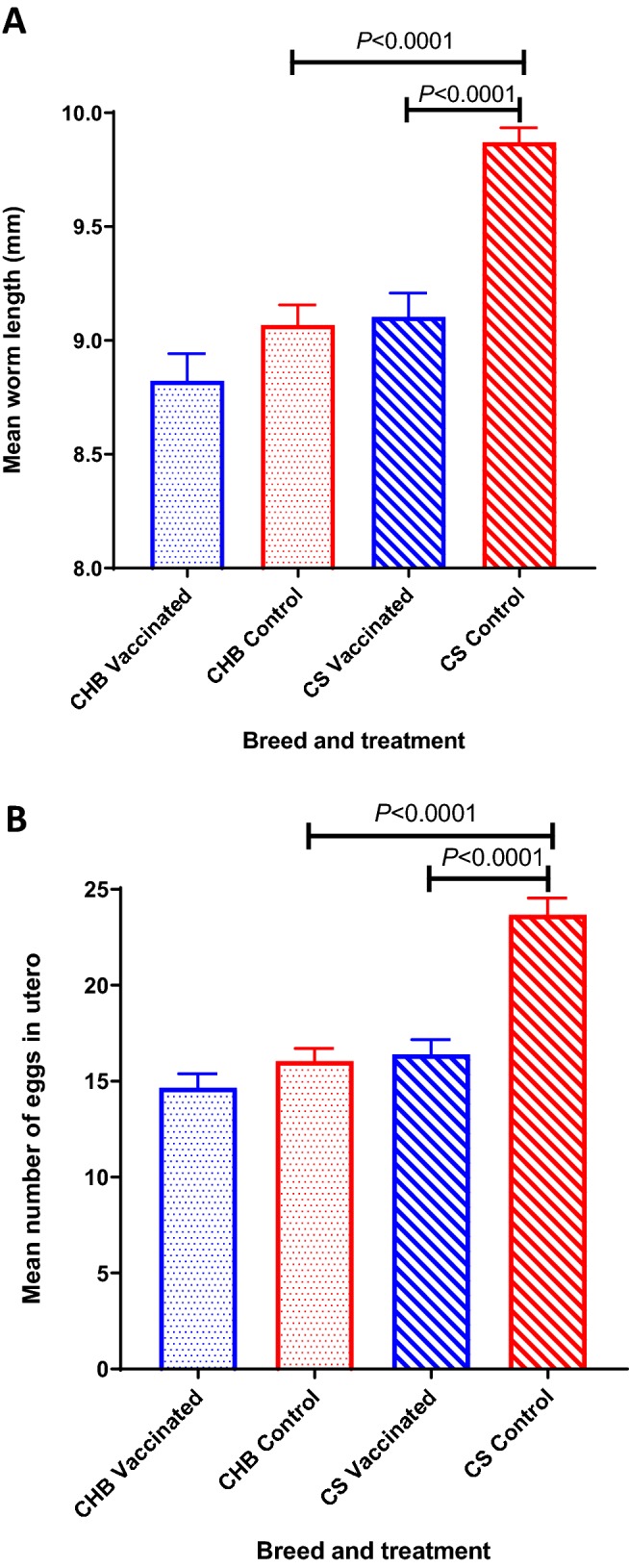
**Effects of immunization of two native sheep breeds from Gran Canaria with recombinant antigens derived from** ***T. circumcincta*** **on worm length and egg production.** Worm lengths (**A**) and the number of eggs in utero in female worms (**B**) are shown for sheep (CS = Canarian sheep; CHB = Canarian Hair Breed sheep) challenged with 2000 *T. circumcincta* L3 three times per week for 4 weeks following immunization with an 8-protein cocktail in the context of Quil A (CS-VAC; CHB-VAC) or with Quil A only (CS-Control; CHB-Control). The mean worm length or mean number of eggs in utero ± SEM is shown (*n* = 193, 284, 339 and 278 for CHB-VAC, CHB-Control, CS-VAC and CS-Control respectively).

Abomasal luminal and mucosal worm burdens (adult and larval stages) were enumerated following standard techniques [9]. The developmental stage (larva or adult) was determined based on length and reproductive structure development. Briefly, 30 adult female nematodes were randomly recovered from each abomasum and measured using a digital photo camera (ProgRes C12^PLUS^) on an inverted microscope (Olympus CKX41) and their eggs in utero (EIU) counted [14]. Several lambs had insufficient worms in the aliquots so, in these cases, all worms were collected from the abomasum and enumerated. Mean worm lengths and numbers of EIU were analysed by one-way ANOVA and the differences between groups identified using Tukey’s multiple comparisons tests. CS vaccinates had similar average burdens at post-mortem (4103 ± 776) to control CS (4410 ± 732) (*p* = 0.796; Figure 4). CHB sheep vaccinates had 33% lower average worm burdens at post-mortem (1892 ± 424) compared to CHB controls (2827 ± 575). This difference was not statistically significant (*p* = 0.329; Figure 4). Comparing burdens between control lambs of the two breeds, CS had, on average, 36% higher worm burdens than CHB sheep; the difference was not statistically significant (*p* = 0.109). More immature worms were observed in the two CHB groups than in the CS groups, with a proportion of 38% and 27% of immature in total worm counts in the vaccinated and control groups of CHB sheep, and 12% and 6% of immatures in the vaccinated and control CS groups. The level of stunting in worms recovered from CHB controls was not significantly different from CHB vaccinates; however, worms from CHB controls were significantly shorter than those recovered from CS controls (*p* < 0.0001). Adult worms recovered from vaccinated CS lambs were significantly shorter than adult worms from the CS control animals (*p* < 0.0001) (Figure 5A). Similarly, CS vaccinates had significantly fewer EIU in female worms retrieved from their abomasa compared to control CS lambs (*p* < 0.0001). Female worms from CHB controls contained significantly fewer EIU than worms from CS controls (*p* < 0.0001) (Figure 5B).

## Discussion

Here, the effect of a *T. circumcincta* prototype vaccine [6, 8] was tested in two breeds of sheep with known differences in their relatively susceptibility to experimental infection with *Haemonchus contortus* [9] and to natural GIN infection in which the predominant genera/species had been identified as *Trichostrongylus* spp., *T. circumcincta* and *H. contortus* [10]. There were two main objectives of the approach taken here: (1) to compare efficacy of the vaccine prototype in breeds of Spanish sheep with data obtained previously for British Texel-cross sheep [6, 8], and (2) to investigate whether the combination of genetic resistance and vaccination would have an additive effect in protection against *T. circumcincta* experimental challenge. Underpinning these objectives was the premise that CHB lambs would be more resistant than CS lambs to experimental infection with *T. circumcincta* larvae. Indeed, this was the case; when comparing the control groups of the two breeds, statistically-significant lower FEC levels over time, lower cumulative FEC, shorter worm length and fewer EIU were observed in CHB sheep when compared to CS sheep. In addition, sheep in the CHB control group harbored 36% fewer worms than the CS lambs, although the difference was not statistically significant. Genetic resistance to *T. circumcincta* in CHB lambs could be related to host mechanisms that cause a delay in larval development as a higher proportion of juvenile worms were enumerated in the CHB lambs than in the CS lambs at post-mortem. Although variability in *T. circumcincta* resistance has been described between individuals within a breed in several breeds [15–17], there have been few references of differences in resistance to this nematode between breeds [18].

In previous trials using this vaccine in Texel-cross lambs, significant differences between vaccinated and non-vaccinated control sheep were observed in both worm burden and FEC over time as well as in cumulative FEC [6]. In the work described here, FEC and worm burden parameters were reduced in vaccinated CS lambs, but the differences were not statistically significant, however, worm length and the number of eggs in female worm uteri were significantly lower in vaccinated CS lambs compared to non-immunised CS lambs. Worm length was not affected in vaccinated Texel-cross lambs [6], suggesting that mechanisms of protection induced by the vaccine, or timing of the response, may be different between breeds. Analogous to this observation, it has been reported that during GIN infection, some breeds of sheep are able to immunologically respond earlier than others [18] and different types of breed responses have been observed [14, 19]. These differences during parasite exposure may be relevant in the vaccine-induced response in each breed of sheep.

In CHB lambs, although vaccinates had lower FEC over time and cumulative FEC, lower worm counts, and their nematodes were shorter, with fewer EIU than observed in the control CHB group, the differences were not statistically significant. Therefore, although there was some evidence that the vaccine may induce a protective effect in this breed, the high level of inherent resistance in CHB lambs of this age made demonstration of the additive or synergistic effects of vaccination less clear. When comparing data from the CHB vaccinates to the CS control sheep, significant differences in all parasitological parameters were observed; such interactions between breed and vaccination may allow better integrated control of GIN and suggest the potential for combining these approaches in an integrated strategy to helminth control [5, 12]. Identifying specific mechanisms of the effector response and discovering why each breed appears to behave differently using the same vaccine and challenge protocol may help inform formulation and delivery to improve the vaccine by stimulating more appropriate immune responses. Future studies will be designed to address this hypothesis.

## Abbreviations

AUC: area under the curve
CHB: Canaria Hair Breed
CS: Canaria Sheep
EIU: eggs in utero
EPG: eggs per gram
FEC: faecal egg counts
GAMM: generalised additive mixed models
GIN: gastrointestinal nematodes
*H. contortus*: *Haemonchus contortus*
L3: third stage larval
M: molar
MTci2: UK derived *T. circumcincta* strain
PBS: phosphate buffered saline
SEM: standard error of the mean
*T. circumcincta*: *Teladorsagia circumcincta*
Tci-APY-1: calcium-dependent apyrase-1
Tci-ASP-1: activation-associated secretory protein-1
Tci-CF-1: cathepsin F-1
Tci-ES20: 20 kDa protein of unknown function
Tci-MEP-1: astacin-like metalloproteinase-1
Tci-MIF-1: macrophage migration inhibitory factor-1
Tci-SAA-1: an homologue of a protective antigen from *Ancylostoma caninum*
Tci-TGH-2: TGF homologue
